# DIGITAL STORIES FROM AN INTERGENERATIONAL, TELEPHONE-BASED REMINISCENCE PROGRAM

**DOI:** 10.1093/geroni/igad104.0790

**Published:** 2023-12-21

**Authors:** Noelle Fields, Ling Xu, Brooke Troutman, Kathryn Daniel, Megan Westmore, Jessica Cassidy

**Affiliations:** The University of Texas at Arlington, Arlington, Texas, United States; The University of Texas at Arlington, Arlington, Texas, United States; Air Force Academy, Air Force Academy, Colorado, United States; University of Texas at Arlington, Arlington, Texas, United States; University of Texas at Arlington School of Social Work, Arlington, Texas, United States; University of Texas at Arlington, Arlington, Texas, United States

## Abstract

Reminiscence strategies combined with an intergenerational approach may yield social and mental health benefits for older adults with cognitive impairment. There is also evidence to support the use of digital storytelling (DST) with persons with cognitive impairment combined with intergenerational programs. DST typically involves the production of a two-to-five-minute audio-visual clip combining text, images, music, photographs, voice-over narration, and other audio. However, there are few studies that combine reminiscence, DST, and an intergenerational approach with persons with cognitive impairment. The current study is a thematic analysis of DST products created by university students in collaboration with older adults with cognitive impairment (N = 27) as part of a larger project examining how intergenerational reminiscence using DST may improve the social and emotional well-being of persons with memory loss. After the completion of a structured, six-week, telephone-based reminiscence, the younger and older adults co-created a storyboard and script as well as selected photos and music to accompany the narrative in the DST. An interactive team approach using the phases of thematic analysis of the DST products resulted in six themes: 1) family, 2) religion and purpose in life, 3) loves and hates, 4) career/work, 5) stress and coping, and 6) major life turning points. Sub-themes included childhood and early adulthood, family legacy, faith, volunteering, travel, music, and resilience. Findings from the study extend current research by examining intergenerational connections, reminiscence, and the use of DST with persons with memory impairment. We close with recommendations for practice and research.

